# Common genetic basis for canopy temperature depression under heat and drought stress associated with optimized root distribution in bread wheat

**DOI:** 10.1007/s00122-015-2453-9

**Published:** 2015-02-24

**Authors:** R. Suzuky Pinto, Matthew P. Reynolds

**Affiliations:** 1 Australian Centre for Plant Functional Genomics, University of Adelaide, Waite Campus, Glen Osmond, SA 5064 Australia; 2 CIMMYT, Int. Apdo. Postal 6-641, 06600 Mexico, DF Mexico

## Abstract

**Key message:**

**QTL related to cooler canopy temperatures are associated with optimal root distribution whereby roots proliferate at depth under drought or near to surface under hot, irrigated conditions.**

**Abstract:**

Previous research using a bread wheat RIL population of the Seri/Babax cross showed that common QTL were associated with cooler canopies under both drought and heat-stressed conditions. A subset of RIL was grown under water-limited and hot-irrigated field environments to test how cooler canopies are related to root development. Eight sisters and the two parents were used in the study with genotypes grouped as COOL or HOT according to their respective QTL for canopy temperature and previous phenotypic data. Root mass production and residual available soil moisture were measured around anthesis at four depth profiles (from 0 to 120 cm depth). When considering different root profiles, there was a clear interaction of QTL with environment. Under water stress, the COOL genotypes showed a deeper root system allowing the extraction of 35 % more water from the 30–90 cm soil profile. The strategy under heat was to concentrate more roots at the surface, in the 0–60 cm soil layer where water was more available from surface irrigation. Since COOL genotypes showed better agronomic performance, it can be concluded that their QTL are associated with more optimal root distribution in accordance with water availability under the respective stresses. The study demonstrates the importance of root development under both water-limited and hot-irrigated environments, and shows a common genetic basis for adaptation to both stresses that appears to be associated with sensitivity of roots to proliferate where water is available in the soil profile.

## Introduction

While the focus of most research in plants is on the above ground organs, the radicular system represents a high proportion of the total plant’s mass and energy requirement. Nonetheless, a comprehensive understanding of root mechanisms involved in, for example, drought and heat response, are imperative to the effort of increasing adaptation of crops to harsher environments under climate change. Roots have a range of functions including anchorage, mechanical support, nutrient and water uptake, and signaling. Roots are also extremely sensitive to water deficit and high temperatures; for example, they show a narrow range of optimum growth temperature compared to other organs (Porter and Gawith 1999).

Under high temperature field experiments, root growth was observed to be diminished due to a reduction in the carbon partitioned below ground, and the number, length and diameter of roots are especially affected if the heat occurs during the reproductive stage (Batts et al. 1998). Drought has different effects depending on the severity. If a moderate drought occurs root development can be promoted because an increased amount of carbon assimilates is sent to the roots; primary root development is increased while lateral roots are repressed (Smith and De Smet 2012). Under drought, high concentrations of ABA can be detected in the roots which have been linked to plant signaling, resulting in stomatal closure and even seed abortion (Prasad et al. 2008). Drought and heat stress symptoms above ground -such as smaller organs and tissue chlorosis- are relatively easy to detect. Nonetheless, relatively few studies have considered the role roots play in stress response mainly because precise, well-controlled, field experimental procedures are not straightforward. As a result many researchers opt for studies in controlled environments where rooting volume and temperatures are generally poorly representative of field growing conditions (Anderson 1986). Molecular control of root development has been studied in Arabidopsis (Larkindale et al. 2005) but in cereals relatively little is known. In maize and rice, mutants have been used to study the lateral root development and crown root elongation. The proteomics of the roots of two *Agrostis* grass species exposed to moderated (30 °C) and intense (40 °C) heat stress were studied by Xu and Huang (2008) showing more proteins associated with stress response mechanisms were up-regulated in the thermotolerant species.

In the field, it has been shown that bread wheat genotypes that invest significant resources in deep root development are capable of extracting residual moisture when drought stress occurs (Reynolds et al. 2007; Lopes and Reynolds 2010). Under heat stress, well-watered plants increase their transpiration rate due to high vapor pressure deficit which permits evaporative canopy cooling. To match evaporative demand requires increased stomatal conductance (Amani et al. 1996) and adequate vascular capacity including in the roots. Some traits can be used as surrogates for the analysis of root development, for example, the measurement of the canopy temperature. Cool canopy temperatures have been associated with increased plant access to water as a result of deeper roots (Lopes and Reynolds 2010). These authors found that genotypes with cooler canopy temperatures reported 30 % more yield associated with an increase of 40 % in root dry weight at 60–120 cm. Genomic regions (QTL) associated with canopy temperature have been co-located with regions controlling other drought adaptive traits including kernel number, grain yield and chlorophyll content (Pinto et al. 2010; Diab et al. 2008; Olivares-Villegas et al. 2008). In a previous study, Pinto et al. (2010) identified 15 QTL for canopy temperature (CT) in the Seri/Babax bread wheat population grown under drought, hot-irrigated and non-stressed conditions. The authors demonstrated five consistent QTL (1Ba, 2Ba, 3Bb, 4Aa, 7Aa) associated with cooler canopies that were common to both drought and heat environments. Three of the QTL were specific only for drought and heat stress, and the other two were also found under non-stressed conditions. The five QTL for CT explained an average of 7 and 14 % of variance under drought and heat, respectively, with maximum of 27.6 % under the heat environment in the 4A-a linkage group. On the same linkage group, a QTL explained a maximum of 27.4 and 17.1 % of yield variation under drought and heat, respectively. The involvement of roots was inferred since cooler canopies are a result of higher transpiration rates which require adequate access to water. For the current study, lines showing contrast in these five QTL for CT were used for the selection of the sisters together with phenotypic data for CT and yield. These five QTL overlapped with QTL for traits previously associated with drought and heat tolerance including water soluble carbohydrates (WSC), kernel number, yield and plant greenness (Kuchel et al. 2007; Marza et al. 2006; Rebetzke et al. 2008).

The importance of roots in determining yield under stressed environments was highlighted by the recent release of rice varieties with improved performance under drought as a result of deep root development achieved through MAS for QTL associated with root length (Steele et al. 2006). The five QTL used in the current study are regions reported in the literature to be associated with root-related traits. A number of QTL for early root length have been mapped in the 1B region of wheat and in its homoeologous region in rice, both crops grown in hydroponic culture (Price and Tomos 1997; Ren et al. 2012). Chromosome 2 was found to be associated with one or more architectural characteristics of seminal roots of durum wheat grown in gel chamber, including length, number and thickness (Sanguineti et al. 2007) and up to 68 % of variance for root length of bread wheat was explained by a QTL identified in this region (Ren et al. 2012). Also, in field grown rice a QTL located in the homoeologous chromosome (Chromosome 8, Ahn et al. 1993) to 2B explained more than 30 % of variance for root thickness under drought stress (Champoux et al. 1995). It seems that the 2B chromosome might contain genes for evapotranspiration efficiency since wheat experiments in pots under controlled conditions (Ehdaie and Waines 1997) showed several QTL in the long arm of the chromosome 2B of bread wheat associated with biomass production––including roots, shoots and spikes. Similarly, chromosomes 3 and 7 of wheat are associated with deep root development and root thickness and Champoux et al. (1995) report QTL in homoeologous regions of rice controlling these traits under field drought-stressed conditions. Up 30 % of the root length variance (Price and Tomos 1997) was explained by a QTL located in chromosome 11 of rice that was hydroponically grown, which maps with segments of chromosomes 4, 5 and 7 of wheat where QTL for CT, NDVI, yield and grain number were previously mapped in field experiments with the Seri/Babax population (Pinto et al. 2010). The genomic region of chromosome 7 seems to contain several genes associated with drought tolerance in wheat, rice and barley where QTL have been identified for osmotic adjustment and related traits in wheat (Morgan and Tan 1996) and it homoeologues in rice (Zhang et al. 2001; Lilley et al. 1996) and barley (Teulat et al. 1998). Using the subset of sisters grouped according to their phenotype and genotypic data in COOL and HOT canopies, the current study was established with the following objectives: (i) to characterize a subset of contrasting Seri/Babax sisters in their agronomic and physiological performance when grown under drought and heat stresses, (ii) to verify the potential of the previously identified QTL in marker assisted selection, and (iii) to test the hypothesis that optimal root distribution provides a common physiological response for adaptation under both drought and heat stress.

## Materials and methods

### Germplasm

The recombinant inbred lines used in this study came from the cross of parents, Seri M82 and Babax (also named *Baviacora M 92* or *Bav 92*), both spring wheat (*Triticum aestivum* L.) semi-dwarf lines with moderate tolerance to drought and heat stress (Olivares-Villegas et al. 2007) and high-yield potential. Only Seri M82 carries the T1BL.1RS (rye) translocation from Kavkaz (Villarreal et al. 1998). The population was constructed for mapping of complex traits and therefore shows a relatively narrow range in phenology and height which is useful to avoid the confounding effect of major flowering and *Rht* genes (Pinto et al. 2010). Ten genotypes were included in this study and classified in two groups: COOL and HOT. The list of eight sisters and two parents is presented on Table 1, including their group (COOL/HOT) and the CT QTL used for the classification.

**Table 1:** List of eight sisters and the two parents (Genotypes 9 and 10) selected from the Seri/Babax bread wheat population for their contrasting phenotypic and genotypic performance under drought and heat stress

Genotype	Cross	Selection History	Group	QTL for which was selected^a^
1	Bav92/Seri	CMSS96Y04084S-0Y-1B-81TLA-0B-0Y-71B-0Y-0Y	COOL	lBa, 2Ba, 3Bb, 7Aa
2	Bav92/Seri	CMSS96Y04084S-0Y-1B-131TLA-0B-0Y-118B-0Y-0Y	COOL	lBa, 2Ba, 3Bb, 4Aa, 7Aa
3	Seri/Bav92	CMSS96Y04051S-0Y-1B-46TLA-0B-0Y-23B-0Y-0Y	COOL	lBa, 2Ba, 3Bb, 4Aa, 7Aa
4	Seri/Bav92	CMSS96Y04051S-0Y-1B-46TLA-0B-0Y-24B-0Y-0Y	COOL	lBa, 2Ba, 3Bb, 7Aa
5	Bav92/Seri	CMSS96Y04084S-0Y-1B-52TLA-0B-0Y-50B-0Y-0Y	HOT	2Ba, 3Bb, 4Aa, 7Aa
6	Bav92/Seri	CMS S 96Y04084S -0Y-1B-72TLA-0B-0Y-62B-0Y-0Y	HOT	lBa, 2Ba, 3Bb, 7Aa
7	Bav92/Seri	CMSS96Y04084S-0Y-1B-93TLA-0B-0Y-103B-0Y-0Y	HOT	lBa, 2Ba, 3Bb, 4Aa, 7Aa
8	Bav92/Seri	CMSS96Y04084S-0Y-1B-131TLA-0B-0Y-117B-0Y-0Y	HOT	IBa, 3Bb, 4Aa, 7Aa
9	Seri M 82	CM33027-F-15 M-500Y-0 M-87B-0Y-0 MEX	HOT	IBa, 3Bb, 7Aa
10	Baviacora M 92	CM92066-J-0Y-0M-0Y-4 M-0Y-0MEX-48BBB-0Y	COOL	IBa, 3Bb

^a^ Indicates the linkage group where the QTL for CT was identified by Pinto et al. (2010)

The HOT genotypes generally carried the Seri allele on those regions where any of the five QTL for CT was identified. The allele from Seri accounted for the undesirable expression of high CT and decreased yield (Pinto et al. 2010). In contrast, the COOL genotypes generally carried the Babax allele on the selected QTL for CT, allele responsible for lower canopy temperatures and high yields. For the QTL × E analyses a factorial design was applied using SAS Proc Mixed.

### Description of the environments and experiments

Two drought and two heat experiments were established in the Yaqui Valley, NW Mexico during 2008–2009 and 2010–2011 seasons. Climatic conditions for the period of experiments are presented on Table 2. Drought trials received approximately 200 ≤ 300 mm of water during the whole cycle, including irrigations and precipitation. Genotypes were sown in November and most water was received before the booting stage. Subsequently, there was moderate drought stress during booting/heading and gradually intensifying stress during grainfilling. To establish the heat experiments, the sowing date was delayed by approximately, 3 months. The air temperature became higher as the cycle progressed, reaching average daily maxima of nearly 37 °C during grainfilling. The heat-stressed trials were fully irrigated every 2 weeks to minimize water limitations that could confound the results. Additionally, the sisters were sown during 2 years under high yielding conditions with minimal water and temperature limitations as control trials. Pest and diseases were controlled during the season in all the experiments. The four trials were established in a complete randomized block design with four replications. Each experiment consisted of ten genotypes sown in double raised beds of 3.5 × 0.8 m using a seed density of 13 g/m^2^. The soil at the Yaqui Valley is classified as sandy-clay and hyposodic vertisol, smectitic chromic haplotorrert according to the World Reference Base (Verhulst et al. 2009).

**Table 2:** Weather conditions for each drought (Drt) and Heat trial sown during 2008–2009 and 2010–2011 crop seasons in the Yaqui Valley, NW Mexico Weather data by stage is summarized using the average of daily records for maximum air temperature (*T*_max_), minimum air temperature (*T*_min_), sum of precipitation (Rain) and sum of evapotranspiration (Eto), according to data from the Mexican National Water Commission (CNA)

Environment	Irrigation (mm)	Year of harvest	Stage	*T*_max_ (°C)	*T*_min_ (°C)	Rain (mm)	Eto (mm)
Drought	<300	2009	Emergence to anthesis −15d	26.2	9.5	2	118
			Anthesis −15d to anthesis +10d	26.4	7.4	0	66
			Anthesis +10d to maturity	29.3	11.0	3	81
		2011	Emergence to anthesis −15d	25.9	6.3	2	157
			Anthesis −15d to anthesis +10d	27.7	7.5	0	83
			Anthesis +10d to maturity	30.7	9.5	0	89
Heat	>700	2009	Emergence to anthesis −15d	29.1	10.6	3	125
			Anthesis −15d to anthesis +10d	31.2	11.6	0	137
			Anthesis +10d to maturity	36.6	15.8	0	153
		2011	Emergence to anthesis −15d	30.6	9.6	0	136
			Anthesis −15d to anthesis +10d	32.2	12.4	0	153
			Anthesis +10d to maturity	36.1	12.3	0	188

The “Irrigation” column indicates the estimated total millimeters of water applied in the whole cycle

*d* Days

### Measurements

Agronomic and physiological measurements were performed on all ten genotypes sown in each environment. For the residual available soil moisture (RASM) and root biomass analyses at heading stage, a subset of four genotypes were selected, two of them COOL and two HOT. Only genotypes number three, four, five and six (Table 1) were included in the root and soil analyses. Traits recorded in the complete pool of genotypes were: yield (g/m^2^), aboveground biomass at maturity (g/m^2^), stem number at anthesis and maturity (stems/m^2^), phenology, water soluble carbohydrates content in the stems at heading ±7 days (%) and canopy temperature during grainfilling (CTg) and vegetative stage (CTv). These measurements were performed using standard protocols cited by Reynolds et al. (2007). Days to heading and days to maturity were determined when 50 % of the plot exhibited 50 % of the spike (Zadoks 5.5) and when 50 % of the plot lost greenness, respectively. For the residual available soil moisture and root biomass analyses, an hydraulic soil corer (Giddings Corp. Co., Fort Collins, CO, USA) as cited by Lopes and Reynolds et al. (2010) was used to extract the soil sample from 0 to 120 cm depth. Sampling was done exactly above the row of plants to obtain soil and root biomass. Soil sampling was performed at heading time plus 10 days (±2 days). On each plot two and four points were sampled in the 2009 and 2011 seasons, respectively. Soil samples were separated into four depth profiles: 0–30, 30–60, 60–90 and 90–120 cm using plastic bags to avoid soil moisture losses before weighing. In the same plot the two/four subsamples were bulked in a single plastic bag according to the corresponding profile. Samples were kept in the field in a cool box. At the research station, the soil was mixed, then, a weighed sample about 100 g (fresh weight) was dried in the oven at 75 °C for 24 h to determine residual soil moisture. The remaining soil was washed and sieved to obtain root tissue. Roots were dried and weighed to determine root biomass production by soil profile.

A student’s *T* test was used to compare the two groups of genotypes and determine differences between COOL and HOT genotypes. The statistical analyses were performed using SAS v9.0. Root biomass and RASM data was standardized by the yearly average (SMean). These relative values for root and RASM used to compare between groups were calculated dividing individual data point by the trial mean.

## Results

### Agronomic and physiological performance of two contrasting groups of sisters

Means for agronomic and physiological traits are presented on Table 3. Differences in CTv and CTg from a previous study (Pinto et al. 2010) were used together with QTL data, to group the lines in two contrasting sets of COOL and HOT genotypes (Table 1). Under drought the two groups reported significant differences of 1.4 and 0.6 °C in CTv and CTg, respectively. In the heat experiments the differences between the two groups were 1.0 and 0.8 °C for CTv and CTg (Table 3). The COOL genotypes yielded 19 and 12 % more than the HOT genotypes under drought and heat, respectively, which was in agreement with 20 % higher biomass production at maturity under drought and 12 % higher biomass under heat. The number of stems was around 20 % higher in the group of COOL genotypes (data not shown). While the growing cycles for drought and heat were on average 112 and 83 days, respectively, the differences in days to heading and maturity between COOL and HOT groups was no more than 3 days in any environment (Table 3). Under drought, the COOL genotypes had 65 % more grains per square meter and 15 % less WSC in the stems but no differences were found for kernel weight in any environment (data not shown); when grown under heat stress the difference in grain number was 20 % more grains produced by the COOL and 40 % less WSC. High and significant correlation with yield was found for canopy temperature measured during the grainfilling stage, biomass at maturity, and grain number in the two environments (Table 3). Under non-stressed conditions both groups of genotypes reported statistically equal plant height differing only in four cm (data not shown).

**Table 3:** Means for COOL and HOT genotypes for 2 years of experiments (2008–2009 and 2010–2011) with Seri/Babax bread wheat grown under drought and heat stress

Trait	Env.	Group mean (SE)			*r*
		COOL	HOT	Pr > |t|	
Canopy temperature during vegetative stage (°C)	Drt	23.5 (0.37)	24.9 (0.50)	0.040**	ns
	Heat	27.0 (0.16)	28.0 (0.17)	0.000***	ns
Canopy temperature during grainfilling stage (°C)	Drt	26.1 (0.11)	26.7 (0.10)	0.001***	−0.70 ***
	Heat	31.6 (0.12)	32.4 (0.24)	0.0055***	−0.75 ***
Yield (g/m^2^)	Drt	204 (10.7)	172 (5.7)	0.017**	–
	Heat	240 (6.9)	215 (4.9)	0.010**	–
Biomass at maturity (g/m2)	Drt	525 (24.4)	440 (10.2)	0.007***	0.90 ***
	Heat	565 (20.2)	505 (11.6)	0.021**	0.84 ***
Heading (dae)	Drt	79 (0.58)	77 (0.77)	0.035**	−0.78 ***
	Heat	51 (0.25)	50 (0.42)	0.003***	ns
Maturity (dae)	Drt	112 (0.85)	109 (0.56)	0.018**	−0.65 ***
	Heat	83 (0.21)	82 (0.48)	0.007***	0.58 ***
Number of grains (†) (grains/m^2^)	Drt^(‡)^	8,985 (725)	5,438 (605)	0.007***	0.93 ***
	Heat	8,380 (365)	6,885 (349)	0.011**	0.70 ***
Water soluble carbohydrates (†) (%)	Drt^(‡)^	32.7 (0.30)	38.6 (1.1)	0.016**	ns
	Heat	9.0 (0.67)	15.4 (2.0)	0.018**	ns
Root: shoot at anthesis	Drt	0.32 (0.02)	0.28 (0.02)	0.018**	nc
	Heat	0.30 (0.04)	0.27 (0.03)	ns	nc

Data for the canopy temperature during vegetative and grainfilling stages was taken from: Pinto et al. (2010) and used for the selection of sister lines included in the current study. Statistically significant values according to Student’s *t* test at levels * α = 0.1, ** *α* = 0.05 and *** *α* = 0.01. All traits were recorded in the complete set of ten genotypes except by those indicated by (†) which were measured in the subset selected for root and RASM analyses.^ (‡)^ Data for 1 year under Drt. Phenotypic correlation (Pearson) is shown as *r* for all the traits with yield using raw data for two replications and 2 years in each environment

*Env* environment, *SE* Standard error of means indicated in brackets, *ns* not significant, *nc* not calculated

### Differences in radicular biomass and residual available soil moisture of the COOL and HOT genotypes

**Fig. 1:**
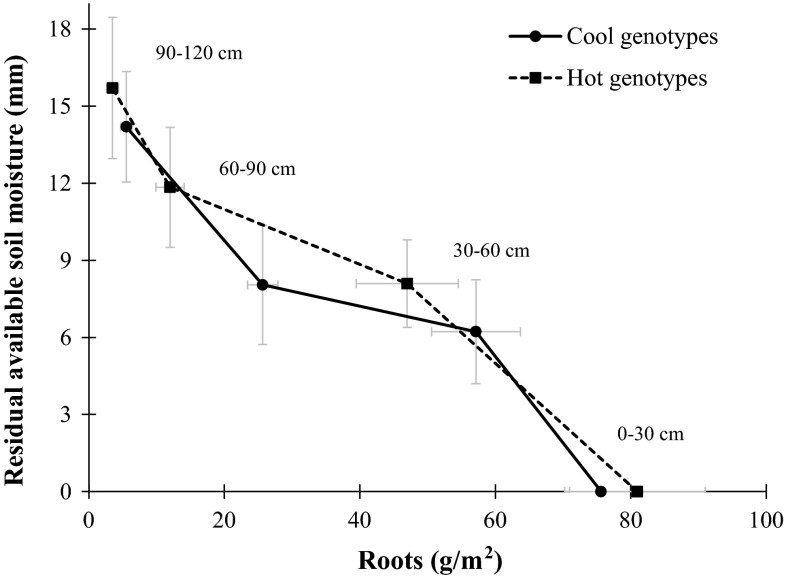
Average of 2 years of experiments for root development and residual available soil moisture at heading +10 days in Seri/Babax bread wheat grown under drought conditions NW Mexico during 2008–2009 and 2010–2011 seasons

**Fig. 2:**
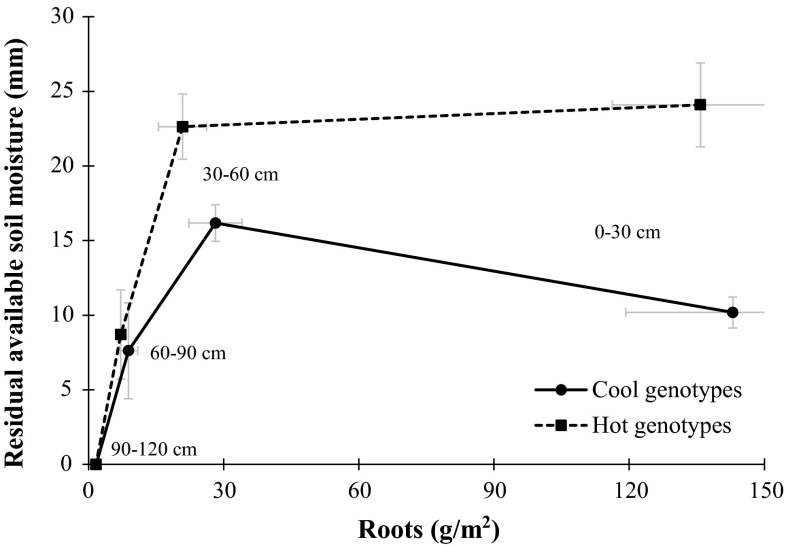
Average of 2 years of experiments for root development and residual available soil moisture at heading +10 days in Seri/Babax bread wheat grown under heat conditions, NW Mexico during the 2008–2009 and 2010–2011 seasons

Significant differences were found between COOL and HOT genotypes for root biomass production and residual soil moisture (Table 4), under both drought and heat stresses, with smaller amounts of residual moisture and more extensive roots generally associated with cooler, higher biomass plants (Figs. 1, 2).

**Table 4:** Significance obtained from the Student’s *t*-test for the standardized means of the COOL and HOT genotypes in 2 years of experiments (2008–2009 and 2010–2011) in Seri/Babax bread wheat grown under drought and heat stress

Trait	Drought			Heat		
	SMean (SE)		Pr > |t|	SMean (SE)		Pr > |t|
	COOL	HOT	*T* test	COOL	HOT	*T* test
Root development (g/m^2^)						
Total roots (0–120 cm)	1.05 (0.054)	0.927 (0.067)	ns	1.046 (0.046)	0.966 (0.070)	ns
Superficial roots 0–30 cm	0.98 (0.082)	1.020 (0.104)	ns	1.031 (0.050)	0.968 (0.080)	ns
Roots 30–60 cm	1.12 (0.065)	0.883 (0.081)	0.0409	1.150 (0.029)	0.849 (0.056)	0.0003
Roots 60–90 cm	1.35 (0.112)	0.598 (0.084)	0.0002	1.142 (0.179)	0.858 (0.120)	ns
Roots 90–120 cm	1.22 (0.193)	0.750 (0.181)	ns	0.993 (0.069)	1.005 (0.285)	ns
Roots 0–60 cm	1.03 (0.057)	0.968 (0.059)	ns	1.048 (0.043)	0.952 (0.068)	ns
Roots 0–90 cm	1.05 (0.050)	0.939 (0.055)	ns	1.051 (0.043)	0.949 (0.070)	ns
Roots 30–90 cm	1.16 (0.065)	0.823 (0.052)	0.0018	1.139 (0.049)	0.861 (0.056)	0.0022
Roots 30–120 cm	1.16 (0.070)	0.788 (0.054)	0.0018	1.150 (0.041)	0.888 (0.053)	0.0031
Roots 60–120 cm	1.28 (0.119)	0.626 (0.098)	0.0016	1.154 (0.157)	0.885 (0.105)	ns
RSM: Residual available soil moisture (mm)						
Total RASM (0–120 cm)	0.798 (0.114)	1.098 (0.068)	0.043	0.806 (0.102)	1.162 (0.106)	0.041
Superficial RASM 0–30 cm	0 (0)	0 (0)	nc	0.609 (0.046)	1.391 (0.145)	0.001
RASM 30–60 cm	0.592 (0.180)	1.551 (0.494)	ns	0.822 (0.061)	1.151 (0.112)	0.032
RASM 60–90 cm	0.704 (0.206)	1.131 (0.082)	0.066	1.22 (0.461)	0.931 (0.272)	ns
RASM 90–120 cm	0.898 (0.093)	0.962 (0.054)	ns	0 (0)	0 (0)	nc
RASM 0–60 cm	0.592 (0.180)	1.551 (0.494)	ns	0.739 (0.045)	1.218 (0.116)	0.006
RASM 0–90 cm	0.660 (0.166)	1.290 (0.169)	0.021	0.806 (0.102)	1.162 (0.106)	0.041
RASM 30–90 cm	0.660 (0.166)	1.290 (0.169)	0.021	0.846 (0.127)	1.132 (0.133)	ns
RASM 30–120 cm	0.798 (0.114)	1.098 (0.068)	0.043	0.846 (0.127)	1.132 (0.133)	ns

*SMean* standardized means or relative values of roots and RASM which were obtained dividing each individual data point by the trial mean (year × environment), *SE* standard error in brackets, *ns* not significant, *nc* not calculated

#### Drought

Root mass measured shortly after anthesis and plotted against residual moisture at the same stage (Fig. 1) shows that COOL genotypes used more of the available water in deeper soil profiles (Table 4, *p* = 0.02 for RASM at 30–90 cm and *p* = 0.04 for RAMS at 30–120 cm), and that root mass was also higher in these two regions (*p* = 0.0018 for both, 30–90 and 30–120 cm). It was observed that the moisture at 0–30 cm was close to zero as a result of the larger concentration of roots in this region and soil exposure that allowed evaporative losses. The total residual soil moisture of the COOL genotypes were significantly different (*p* = 0.043) from that of the HOT genotypes, with the COOL genotypes leaving 25 % less water in the whole (0–120 cm) soil profile around anthesis.

#### Heat

Measurements of root development and RASM shortly after anthesis in the heat experiments showed that the HOT genotypes left more residual soil moisture across the whole soil profile (Table 4, *p* = 0.04) down to 120 cm (Fig. 2). The strongest contrast was found nearer the surface at profiles 0–30 and 30–60 cm (Fig. 2) where the COOL genotypes left 60 % (*p* = 0.001) and 30 % (*p* = 0.032) less moisture than the HOT genotypes, respectively. This result was consistent with the COOL genotypes having relatively more superficial roots than deep roots compared to the HOT genotypes. For example, in the 30–60 cm region the COOL genotypes developed 35 % more roots (*p* = 0.0003) than the HOT genotypes.

#### Roots and RASM partitioning under heat and drought

**Fig. 3:**
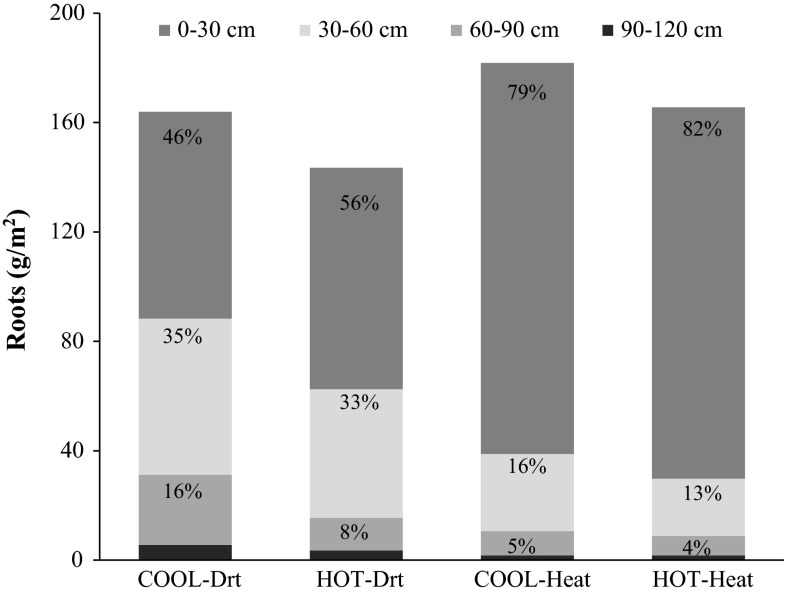
Root distribution across the whole soil profile (0–120 cm depth) in Seri/Babax bread wheat is presented as the average of 2 years for each environment. *T*-tests revealed COOL and HOT genotypes differed significantly for the following profiles: under Drt, 30–60 cm (*p* = 0.04), 60–90 cm (*p* = 0.0002), 30–90 cm (*p* = 0.0013), 30–120 cm (*p* = 0.0018) and 60–120 cm (*p* = 0.0016); under heat, 30–60 (*p* = 0.0003), 30–90 (*p* = 0.0022), 30–120 (*p* = 0.0031). Percentages are the proportion of roots in each profile relative to the total amount of roots produced at 0–120 cm; the proportion in the 90–120 cm profile was <3 % for both groups under Drt and Heat

Comparing the total amount of roots (0–120 cm profile), it was found that the COOL genotypes produced only about 10 % more root tissue than the HOT genotypes under both heat and drought (Fig. 3). However, the analysis of the distribution showed that greater differences were found below 30 cm. Both COOL and HOT genotypes concentrated most of their radicular development (˜ 80 %) in the 0–30 cm profile when grown under heat stress, while under drought they tended to be more equally distributed across the 0–30 and 30–120 regions (Fig. 3). Under drought 54 % of the total root biomass of the COOL genotypes was located in the 30–120 cm profile (Fig. 3), while the remaining 46 % was superficial (0–30 cm). The HOT genotypes showed a smaller proportion (44 %) of roots in the 30–120 cm under drought. The amount of roots found at 0–30 cm under heat, was four times greater than roots from 30–90 and 30–120 cm. Combined analyses across environments and years (QTL × E) showed highly significant interactions between QTL (i.e., COOL v HOT) and the relative distribution of roots across the soil profile which is consistent with the observation that under drought the COOL-QTL favor deeper roots, while under heat stress the COOL-QTL favor more superficial roots (data not shown).

## Discussion

Notwithstanding the well-documented adaptive value of phenological escape (earliness) from drought and heat stress (Barnabás et al. 2008), the potential confounding effect of phenology was avoided in this study by pre-selecting lines of similar heading time (heading range for 2 years averaged 7 and 5 days for drought and hot-irrigated environments, respectively), with contrasting agronomic performance and CT.

### Drought

Phenotypic differences between the COOL and HOT genotypes were consistent with their agronomic performance and root mass and distribution profiles. The results showed that under drought, cooler canopy temperatures were associated with genetic gains of 19 % in yield, 20 % in biomass, and 40 % in deep roots, at 30–90 cm and 30–120 cm (Table 3; Fig. 1). Similar results were found by Lopes and Reynolds (2010) who reported that genotypes with lower canopy temperatures developed 40 % more root mass at 60–120 cm and 30 % higher yields. In the current study, the differences in the root architecture of the two groups were further supported by the amount of residual soil moisture (RASM) at depth. In the superficial layers both groups of genotypes left similar amounts of moisture. However, the analysis of the deeper section from 30–90 and 30–120 cm showed that in these regions the COOL genotypes were able to extract more water leaving 35 and 25 % less residual available moisture than the HOT genotypes (Fig. 1). The capacity of COOL genotypes to extract extra water in the 60–90 cm profile was also associated with an increase of 115 % of root biomass in the same profile under drought (Fig. 1). Lopes and Reynolds (2010) reported that genotypes with greater root development in the deep regions had lower amounts of WSC in the stems, perhaps as a result of more WSC being translocated to the roots to support deep root development. The current study obtained similar results, showing that the WSC content of the COOL genotypes was 15 % lower (Table 3) than the WSC of the HOT genotypes when grown under drought, and 40 % lower under heat. The later was supported by phenotypic correlations with the root distribution under drought, which showed that root biomass in the 60–90 (*r* = −0.54, *p* = 0.057), 60–120 (*r* = −0.68, *p* = 0.015) and 90–120 (*r* = −0.72, *p* = 0.006) soil layers was negatively associated with the percentage of WSC (using raw data) in the stems measured at around anthesis.

### Mechanisms that may be determined by the “COOL QTL”

Drought stress usually promotes hormone signaling; in particular, ABA concentrations are increased in the roots, helping in the maintenance of root growth and water uptake (Prasad et al. 2008). Manschadi et al. (2006) compared two wheat genotypes with different root architecture in root chambers, finding that the root length density below 90 cm in the drought-tolerant variety was almost four times greater than in a standard Australian variety. These authors found that the former showed more compact horizontal root architecture with a narrow angle but greater vertical development. This root pattern allowed superior water extraction capacities of the drought-tolerant variety (~ 25 % more water uptake from 60 to 90 cm). They proved that post anthesis, a drought-adapted variety was able to continue development, focusing in the central and deepest soil layers, in contrast with the standard variety which equally extended its roots horizontally and vertically.

#### Heat

Several studies discuss the relevance of the root development as a key trait for drought tolerance, but scarce information regarding its role under heat stress is available. Deep root development at high temperatures has been associated with higher leaf transpiration rates. Plants with a strong radicular system are able to satisfy the high evaporative demand through elevated transpiration rates under hot irrigated conditions and thus maintain cooler canopies (Amani et al. 1996; Bonos and Murphy 1999). While studies with tall fescue and ryegrass showed that high temperatures generally decreased root dry weight and photosynthetic rate, tall fescue, considered heat-tolerant, exhibited greater root mass at 0–40 cm (Jiang et al. 2001) and a faster depletion of soil water (%). The current study found that the group of COOL genotypes had higher amounts of radicular tissue in all soil profiles down to 120 cm depth, but especially in the 30–60 cm profile. These results showed that under heat stress, cooler canopy temperatures were associated with genetic gains of 12 % in yield, 12 % in biomass and 35 % in root development in the 30–60 cm layer (Fig. 3). When water uptake was studied, the COOL genotypes were more effective in removing soil moisture, resulting in 30 % less RASM at 30–60 cm than the HOT genotypes (Fig. 2). Studies with Kentucky bluegrass showed that the maintenance of transpiration under heat stress was an important attribute for performance under stress. Comparison between heat-tolerant and susceptible cultivars showed that those with canopy temperatures 5 °C cooler had 65 % more roots at 30–45 cm (Bonos and Murphy 1999). At the cellular level, it has been observed that the thermal stability of the plasma membrane of wheat roots is affected by high temperatures (Zhao et al. 2011). Structural analyses of proteins located in the root membranes have shown that above 25 °C the proportion of α-helix and β-sheet changed due to unfolding and disordering of structures. This change in the structure of plasma membrane proteins resulted in the reduction of H^+^-ATPase activity, an enzyme responsible for multiple physiological functions such as nutrient uptake and cell growth, especially under stress conditions (Janicka-Russak 2011).

### Significance of these results to breeding

#### Genetic confirmation that CT is associated with effective root development

Root growth measurement is challenging and usually involves intensive and destructive techniques to obtain root tissue, however, canopy temperature, which is much easier to measure, is associated with the plant’s ability to extract deep water (Reynolds et al. 2007; Lopes and Reynolds 2010) and can be easily measured using infrared technology. Data from a previous study using these lines showed that the genotypes exhibited genetic variation for canopy temperature under heat-stressed growing conditions and that the COOL group had lower temperatures; this was supported by the identification of QTL associated with this trait (Pinto et al. 2010). For example, the QTL for CT at chromosome 2B was classified as stress exclusive (drought and heat) and was also reported as the main QTL responsible for root developmental pattern in wheat, namely the maximum root length of lateral and primary roots (Ren et al. 2010; Sanguineti et al. 2007). Studies with rice and barley reinforced the importance of the QTL utilized herein as regions that might contain genes affecting root architectural characteristic and physiological attributes that determine plant performance (Zhang et al. 2001; Teulat et al. 1998; Champoux et al. 1995). Both parents from the experimental population of this study were identified as differing significantly in yield performance under drought while showing high-yield potential (Reynolds et al. 2000). Their breeding value––as genetic sources of numerous varieties and cultivars––is recognized by breeding programs elsewhere (Fox et al. 1996; IWIS database, CIMMYT Wheat Germplasm Bank). Seri M82 (IWIS CODE: M31 IBWSN S-1 MXI96-97) is a Veery ‘S’-derived variety, susceptible to severe drought (moderately tolerant to moisture stress) (S. Rajaram, pers.comm.; CIMMYT 1986); Babax (IWIS CODE: CM92066-J-0Y-0 M-0Y-4 M-0Y-0 MEX-48BBB-0Y) is a Baviacora variety sister line, tolerant to severe moisture stress (S. Rajaram, pers.comm.; CIMMYT 1986).

Four of the five CT QTL involved in this study showed favorable expression linked to the presence of the Babax allele. Segregation distortion patterns in the 1B chromosome in the Seri/Babax population result in 75 % of the RIL containing the allele from Babax in this region (Mathews et al. 2008). This allele has been reported as responsible for the cool canopies and increased yield in previous studies with the same population (Pinto et al. 2010; Olivares et al. 2007) and other studies have associated the short arm of the 1B chromosome with traits related to transpiration efficiency (Rebetzke et al. 2008). In the subset of RIL included herein, the Seri allele associated with the T1BL.1RS (rye) translocation resulted in negative effects on yield and in warmer canopy temperatures which was in agreement with previous studies involving Seri crosses grown under drought stress and irrigated conditions (Pinto et al. 2008; Mathews et al. 2008; Peake 2003). However, the effect of the T1BL.1RS translocation seems to be environment-dependent (Rattey et al. 2009) since it also has been found to be advantageous for drought adaptation in earlier studies (Villareal et al. 1995). In the 4A chromosome, unfavorable effects from the Seri allele were observed on canopy temperature in a previous study; the presence of the Babax allele in the 4A chromosome of the RIL resulted in cooler canopy temperatures which were apparently associated to larger aboveground biomass and yield increments where as much as 27 % of genetic variance for these traits have been linked to the Babax parental (Pinto et al. 2010). Results from the current study indicated that the COOL genotypes (which generally possessed the Babax allele in the 4A region) showed significantly higher aboveground biomass production as well as higher radicular development.

#### Common QTL for heat and drought

Pinto et al. (2010) showed for the first time common QTL associated with adaptation of wheat to both drought- and hot-irrigated conditions in the Seri/Babax population, and inferred the involvement of roots since cooler canopies were associated with better performance in both environments. The current study, by measuring root growth in subsets of iso-QTL lines from the same population has provided definitive evidence for the involvement of roots. However, the response of roots was not simply to grow deeper or more extensively, but rather to adapt to the specific needs of the environment. Namely, under drought, the roots of the cooler lines showed a greater distribution at depth. On the other hand, under heat stress, the roots of the cooler lines showed a relatively greater proportion of roots at the surface where access to water was more reliable given frequent gravity irrigations in this treatment. This would suggest that the QTL of COOL lines may be exerting their influence at a relatively high level of integration and be involved in determining root distribution pattern in response to environmental cues. This is backed up by work that linked CT to plant growth regulation (Tang et al. 2008; Wardlaw 1974). This selective root performance observed in bread wheat RIL is supported by results from a recent study with *Arabidopsis* which revealed that water availability determines root development, influencing the position of lateral branches and root hairs. The authors indicate that roots can distinguish between soil areas containing air or humidity and are able to respond according to the environment. This kind of response is known as hydropatterning, a conserved process not exclusive to *Arabidopsis* but present also in cereals like Maize and Rice (Bao et al. 2014). In addition, the specificity of the CT QTL previously reported by Pinto et al. (2010) was supported by an interesting trade off observed between stem water soluble carbohydrates (WSC) content and root growth under both stresses. Under drought, root development in the deep soil layers (60–120 cm) was negatively and significantly associated with WSC, while under heat, the negative association with WSC was found in the upper soil region at 30–60 cm. These results were consistent with the findings from Lopes and Reynolds (2010) regarding the possible contribution of stored stem WSC to the development of deeper roots under drought.

## Conclusions

QTL conferring tolerance to both heat and drought stress provide useful opportunities for adapting wheat to climate change, under which both stresses are expected to increase. If one or more of the QTL can be used to derive close markers, they would be especially useful in molecular breeding since heat and drought are both challenging targets separately, and are expected to increasingly occur together (Sanderson 2011). The result also confirms the value of using CT as a proxy for favorable expression of root traits under both heat and drought stress by putting it on a firmer genetic basis. In addition, the observation that these QTL affect adaptive root response gives a useful lead into understanding the genetic basis of how root growth may be regulated.

### **Author contribution statement**

RSP: Conducted all of the experiments that were based on an earlier study she also published. She performed all data analysis and led the write-up. MPR: Designed the experiment and participated in all aspects of data analysis and writing of the paper.
